# Convolutional neural networks: applications, challenges and future prospects in brain tumor research

**DOI:** 10.3389/fneur.2026.1759459

**Published:** 2026-02-23

**Authors:** Peng Zhang, Zhen Yang

**Affiliations:** Department of Neurosurgery, The Second People’s Hospital of Hefei, Hefei Hospital Affiliated to Anhui Medical University, Hefei, China

**Keywords:** brain tumor, convolutional neural network, image segmentation, magnetic resonance, prognosis prediction

## Abstract

As one of the most common malignant tumors in the central nervous system, brain tumors can cause neurological dysfunction and functional impairment. The early precise diagnosis, therapeutic efficacy evaluation, and prognosis prediction of brain tumors are of crucial significance for the formulation of treatment plans and the extension of survival periods for patients. In recent years, artificial intelligence (AI) has been applied in numerous biomedical fields, including the identification, diagnosis, and treatment of brain tumors. Deep learning (DL) is such an AI tool, and convolutional neural networks (CNNs) are widely used deep learning methods. With their powerful capabilities in automatic feature extraction and pattern recognition of images, CNNs have demonstrated great potential in the analysis of medical images of brain tumors. This paper systematically reviews the research progress of CNNs in brain tumors (tumor region identification and segmentation, benign and malignant classification, IDH mutation status prediction, and differentiation of pseudo-progression and recurrence), and deeply analyzes the current challenges and future development directions, aiming to provide a cutting-edge reference for neurosurgeons and researchers.

## 1 Introduction

Brain tumors are a heterogeneous group of common intracranial tumors, leading to significant mortality and morbidity. Malignant brain tumors are among the most aggressive and fatal tumors in all age groups (1). According to the 2021 World Health Organization (WHO) classification of central nervous system tumors, brain tumors are classified into four grades (I to IV), with increasing malignancy and worsening prognosis (2). In fact, in clinical practice, tumor type and grade influence treatment options. Among WHO grade IV tumors, glioblastoma is the most aggressive primary brain tumor, with a median survival of only 12–15 months after diagnosis (3). In recent years, preoperative diagnosis and treatment of brain tumors have mainly relied on imaging examinations, including auxiliary examinations such as computed tomography (CT), magnetic resonance imaging (MRI), positron emission tomography (PET), and ultrasound. Most medical image interpretations are performed by radiologists. Researchers have recognized the necessity of computer-assisted intervention in medical image analysis due to the wide range of pathologies, early lesion missed diagnosis, and inter-individual differences in radiological imaging interpretations by different experts. Deep learning has enhanced the ability to recognize, classify, and quantify medical imaging patterns and helps address this need. Empirical studies have shown that convolutional neural networks (CNNs) can identify hierarchical image features and derive higher-level features from lower-level ones (4). CNNs have demonstrated effectiveness in medical image analysis, including image segmentation, image registration, image fusion, image annotation, computer-aided diagnosis and prognosis, lesion/marker detection, and microscopic imaging analysis. In this review, we aim to explore how convolutional neural networks are revolutionizing the diagnosis and treatment of brain tumors.

## 2 The advantages of convolutional neural networks in medical image analysis

Pathological assessment of tissue samples is the gold standard for tumor diagnosis and grading. However, a non-invasive tool capable of accurately classifying tumor types and inferring their grades would be highly desirable. Although several non-invasive imaging modalities can visualize brain tumors, such as MRI, which conveys macroscopic information about the location, size, extent, characteristics, and relationship with surrounding structures of the lesion. Besides structural information, MRI can also assess microstructural features, such as the structure of tumor cells, microvascular structure, and perfusion. However, they are limited in evaluating microscopic changes of tumors. With the rapid development of artificial intelligence and machine learning technologies, deep learning, especially convolutional neural networks, has become a core technology in the field of medical image analysis. CNN can simulate the hierarchical structure of the human brain’s visual cortex and utilize core modules such as convolution operations and pooling operations to automatically extract features from the original image from low-level to high-level, without the need for manual design of feature extractors, effectively solving problems such as feature engineering relying on professional knowledge and weak generalization ability in traditional machine learning (5). Compared with traditional methods, CNN has advantages in medical image analysis such as automated feature extraction, high recognition accuracy, and wide application scenarios. It can quickly process high-dimensional medical image data, discover subtle features that are difficult for human vision to perceive, and achieve precise localization and classification of lesion areas through large-scale data training and optimization. It can meet the needs of multiple aspects such as diagnosis, efficacy evaluation, and prognosis prediction. These advantages have provided a new technical path for brain tumor medical image analysis.

## 3 The advantages of convolutional neural networks in the field of brain oncology

Convolutional neural networks allow the use of a large amount of data from radiological images and obtain quantitative features of tumor histology and biomarkers in a non-invasive manner, even attempting to predict molecular characteristics and prognosis, and providing more personalized treatment (6). CNN has the potential to improve accuracy and efficiency, and is useful in differential diagnosis in cases that are difficult to compare through MRI assessment between glioblastoma multiforme and primary central nervous system lymphoma. Tangsrivimol et al. (7) used the EfficientNetB4 architecture within the CNN framework to analyze 320 patients with suspected glioblastoma multiforme or primary central nervous system lymphoma who had contrast-enhanced T1-weighted images. Additionally, in the early diagnosis of brain metastases, in stereotactic radiosurgery where a strict understanding of the number and location of metastases is required, the 2D and 3D texture analysis model using the T1-weighted imaging post-contrast sequence can be used to determine whether the tumor is a metastasis of lung cancer, melanoma, or breast cancer, as it can show the differences in the local environment. It is also significant for metastatic lesions and peritumoral edema, for example; Chou et al. (8) studied the automatic identification and quantification of metastatic brain tumors and peritumoral edema based on deep learning neural networks, and the Dice similarity coefficients of the segmentation models for the separation of metastatic tumors and brain edema were 71.6 and 85.1%, respectively. The research results indicate good diagnostic efficacy for the identification of metastatic tumors and peritumoral edema. However, computer-aided design must be used in an appropriate environment because if the sensitivity threshold is too low, there may be overdiagnosis. If the sensitivity threshold is too high, small lesions may not be detected. For traditional surgical treatment of brain tumors, including robot-guided surgery, there are limitations such as the inaccuracy of lesion extension. CNN can provide algorithms to reduce these limitations, precisely delineate the size and location of the lesion, and guide the surgeon in real time.

## 4 The application of convolutional neural networks in brain tumor image segmentation and classification

### 4.1 Tumor region identification and segmentation

The precise identification and segmentation of tumor regions is the foundation of brain tumor diagnosis and treatment, providing crucial basis for tumor staging, treatment plan formulation, and efficacy evaluation. CNN, with its powerful feature extraction and pixel-level classification capabilities, has become the core technology for brain tumor segmentation, and is most widely used in MRI image segmentation (9). MRI is the preferred imaging modality for brain tumor diagnosis, with advantages in multi-sequence imaging (such as T1-weighted, T1-enhanced, T2-weighted, FLAIR, etc.), and different sequences can clearly display the different structural features of the tumor (such as tumor parenchyma, edema area, necrotic area). The CNN model can integrate the complementary information of multi-sequence MRI data to improve segmentation accuracy. For example, 3D U-Net processes multi-sequence MRI three-dimensional volumetric data, fully utilizing spatial information and complementary features between sequences, achieving precise segmentation of tumor core regions, edema areas, and the entire tumor area (10). Additionally, multimodal data fusion strategies (such as combining MRI with PET images) have also been used to improve segmentation performance. PET images can reflect the metabolic activity of the tumor and complement the anatomical structure information of MRI, helping to accurately identify tumor invasion areas. In addition, multi-modal data fusion strategies (such as combining MRI with PET images) are also used to improve the segmentation performance. PET images can reflect the metabolic activity of tumors and complement the anatomical structure information of MRI, which is helpful to accurately identify the tumor invasion area (11–13). Convolutional neural network (CNN) has become the core technical support of brain tumor image analysis by virtue of its powerful feature extraction and spatial modeling capabilities, and has made a breakthrough in segmentation accuracy and classification efficiency. Furthermore, rational use of hidden layers is critical for improving the analytical performance of CNNs in brain tumor imaging. Shallow hidden layers capture low-level visual features, such as tumor edges, texture gradients and local pixel differences, whereas deep hidden layers aggregate and abstract high-level semantic features including tumor heterogeneity, invasion patterns and tissue boundaries between tumors and normal tissues. Cross-layer integration of hidden features, which covers multi-scale feature fusion, residual connections and hidden layer optimization with embedded attention mechanisms, enables CNNs to fully exploit multi-dimensional information from brain tumor images. This strategy effectively reduces gray-scale overlap between tumors and normal brain tissue, and improves detection accuracy for small lesions and regions with blurred boundaries. Reasonable configuration of hidden layer quantity, node scale and connection patterns balances the model feature expression ability and computational efficiency, laying a structural foundation for accurate segmentation at millimeter scale and efficient classification of brain tumors (14, 15). Meanwhile, as a key supplementary strategy, transfer learning has been widely explored to resolve core bottlenecks in brain tumor segmentation with CNNs. By transferring pre-trained universal visual features from large-scale natural image datasets (such as ImageNet) or medical image datasets across different diseases to brain tumor segmentation tasks, it effectively mitigates the scarcity of high-quality annotated data and poor generalization of models with small sample sizes. It adopts two primary implementation approaches: first, fine-tuning pre-trained CNN backbones (such as U-Net, ResNet) to optimize feature decoding specific to tumor; second, transfer learning adaptable to domain variations, which reduces data distribution discrepancies across different centers via adversarial training and directly addresses challenges of data domain shift from multiple centers. Existing studies confirm that transfer learning significantly improves the Dice similarity coefficient of brain tumor segmentation, reduces missed detection rates for small lesions, and achieves synergistic optimization with data fusion using multi-modal information and the inherent feature extraction advantages of CNNs (16, 17). For multi-sequence MRI data, CNN can automatically fuse modal information such as T1, T2, FLAIR, and solve the problem of gray-scale overlap between tumor and normal brain tissue, and provide millimeter-level accurate reference for surgical planning (18, 19). At present, the technology has gradually landed in the clinical auxiliary diagnosis system, but it still needs to break through the challenges such as multi-center data domain migration and missed detection of small lesions (20). In the future, by combining attention mechanism with federal learning, and further integrating optimized transfer learning strategies, CNN will further enhance the generalization ability of segmentation and classification, and become a standardized tool for early screening and accurate diagnosis and treatment of brain tumors.

### 4.2 Classification of benign and malignant brain tumors

The core of the classification of benign and malignant brain tumors lies in differentiating the invasiveness and malignancy degree of the tumors. The CNN model achieves automatic classification by learning the characteristic differences of benign and malignant tumors in the images (such as the clarity of tumor boundaries, enhancement patterns, edema degree, etc.). For example, classification systems based on models like AlexNet and ResNet have achieved an accuracy of over 90% in the classification of benign and malignant tumors through feature extraction and classification of MRI images. Moreover, the CNN model combined with multimodal imaging data (such as MRI + CT) can integrate anatomical structure and density information, further improving the classification performance. In clinical applications, the rapid classification model for benign and malignant tumors can provide rapid diagnostic references for emergency patients and secure time for surgical treatment.

The brain tumor classification model (BCM-CNN) includes advanced three-dimensional (3D) models based on the use of enhanced convolutional neural networks (BCM-CNN). Experimental results show that BCM-CNN achieved the best results due to optimizing the hyperparameters of CNN to enhance its performance (21). In terms of model architecture selection and optimization, researchers designed various efficient CNN models based on the characteristics of brain tumor images. Traditional deep CNN models (such as ResNet, DenseNet) solve the problem of gradient disappearance in deep networks through residual connections and dense connections, and can extract more abstract high-level features, suitable for overall feature analysis of tumors. The ResNet50 model, with a 50-layer network structure and residual block design, demonstrates strong feature representation ability in the discrimination of benign and malignant brain tumors. For example, Ali et al. (22) studied the application of CNN in the classification of gliomas, meningiomas, and pituitary tumors. CNN models (including the Classic layer architecture and ResNet50 architecture) were trained and evaluated using an 80:20 training-test split. The results show that both architectures can accurately classify brain tumors. The accuracy of the classic layer architecture reached 94.55%, while the ResNet50 architecture surpassed it with an accuracy of 99.88%. Compared with previous studies and 99.34%, it proves that CNN, especially the ResNet50 architecture, is effective in brain tumor classification and has great potential in helping medical professionals accurately diagnose and plan treatment. Another study (23) proposed a genetic algorithm (GA)-CNN hybrid for detecting glioblastoma and other intracranial benign tumors. In this method, the appropriate CNN architecture is automatically selected with the help of the genetic algorithm. Researchers were able to correctly identify gliomas, meningiomas, and pituitary tumors in 90.9 and 94.2% of cases.

The application of 3D CNN (3D-CNN) provides spatial dimension features for the discrimination of benign and malignant tumors, especially suitable for processing MRI volumetric data (24). Unlike 2D CNN, which only uses information from a single slice, 3D-CNN extracts features in three dimensions (length, width, and depth) using 3D convolution kernels, can fully capture the morphological features, spatial distribution, and anatomical relationship with surrounding tissues of the tumor in three-dimensional space, avoiding misjudgment due to information loss between slices. Models such as 3D ResNet, 3D VNet, etc. perform well in the discrimination of benign and malignant brain tumors. Some studies have used 3D U-Net to extract features from multi-sequence MRI volumetric data and combined an adaptive weighted fusion strategy, achieving an accuracy of 96.3% in the discrimination between glioblastoma and benign meningiomas, and 91.8% in the discrimination between low-grade gliomas and benign astrocytomas, effectively solving the discrimination problems of some borderline tumors. Furthermore, Mzoughi et al. (25) studied the deep multi-scale 3D convolutional neural network (3D CNN) architecture, using the entire volume T1-Gado MRI sequence to classify glioma brain tumors as low-grade gliomas (LGG) and high-grade gliomas (HGG). The quantitative evaluation conducted through the well-known benchmark (Brats-2018) proved that compared with 2D CNN variants, the proposed architecture generated the most discriminative feature maps to distinguish between LG and HG gliomas. The proposed method achieved a 96.49% overall accuracy on the validation dataset, providing promising results superior to the most recent supervised and unsupervised advanced methods. The same study, Yamashiro et al. (26) developed a 3D CNN model using enhanced T1-weighted images, using the pre-trained Clara segmentation model provided by NVIDIA and the original 2D classification model to implement an automatic glioma grading system. The 3D CNN has good diagnostic value in the classification of gliomas. Zhuge et al. (27) used a deep 3D convolutional neural network to automatically grade traditional MRI images, also obtaining good diagnostic value. In summary, the research indicates that CNN has a high diagnostic value in differentiating intracranial benign and malignant tumors.

### 4.3 Prediction of IDH mutation status in brain tumors

Isocitrate dehydrogenase (IDH) mutations are common genetic mutations in brain tumors, closely related to tumor development, treatment response, and prognosis (28). The prognosis of patients with IDH mutant brain tumors is generally better than that of wild-type patients, and they are sensitive to specific targeted therapies. Therefore, the precise prediction of IDH mutation status is of great significance for the formulation of treatment plans (29, 30). Traditional IDH mutation detection relies on pathological biopsy, which is an invasive operation and has the risk of sampling bias. However, the image-based radiomics method based on CNN can predict the IDH mutation status without invasive imaging data. The CNN model learns the feature differences between IDH mutant and wild-type brain tumors in imaging (such as tumor morphology, enhancement pattern, signal intensity, etc.) to construct a prediction model (31). For example, a CNN model based on MRI T2-FLAIR sequence, by extracting deep features of the tumor area, has an accuracy of IDH mutation status prediction over 80%; a CNN model combining multi-sequence MRI data and clinical features (3D U-Net) can increase the prediction accuracy to over 85%. Additionally, some studies have further improved the prediction specificity and sensitivity by partitioning the tumors (such as the core area and edema area) and extracting features separately and integrating them (32). Currently, CNN still faces some challenges in IDH mutation status prediction: first, there are differences in imaging equipment and scanning parameters among different hospitals, resulting in inconsistent data distribution and affecting the generalization ability of the model; second, the imaging features of some tumors have a weak correlation with the IDH mutation status, making it difficult to extract effective predictive features; third, there is a lack of large-scale, multi-center labeled datasets for model training.

## 5 Convolutional neural network in the differentiation of pseudo-progression and recurrence in brain tumor treatment

The differentiation between pseudo-progression (PsP) and recurrence (TR) after brain tumor treatment is a difficult issue in clinical diagnosis. PsP refers to the imaging enhancement resembling a tumor after radiotherapy, usually occurring within 3–6 months after treatment, and does not require special treatment; while tumor recurrence requires timely adjustment of the treatment plan (33–35). Both PsP and TR show similar appearances on conventional MRI images, and traditional differentiation methods (such as enhanced MRI, PET-CT) have limited accuracy, and PET-CT has the problem of radiation exposure. CNN, with its powerful feature extraction ability, provides a new solution for non-invasive and precise differentiation of PsP and TR. The CNN model learns the potential feature differences between PsP and TR in imaging (such as texture features of the enhanced area, signal uniformity, dynamic enhancement curves, etc.) to achieve automatic differentiation. For example, the CNN model based on dynamic contrast-enhanced MRI (DCE-MRI) can achieve a PsP/TR differentiation accuracy of over 85% by extracting hemodynamic and texture features of the tumor area; the CNN model combining MRI multi-sequence data (such as T1 enhancement, FLAIR, DWI) can integrate multi-dimensional information such as anatomical structure, water molecule diffusion, and inflammatory response, and the differentiation accuracy can reach over 90%. Ying et al. (36) retrospectively investigated 234 patients who underwent radiotherapy after glioma resection and had suspected recurrence lesions detected in follow-up MRI. They used different convolutional neural network (CNN) models to learn the radiological features indicating glioma recurrence and radiation necrosis from MRI scans. In the evaluated CNN models, ResNet10 showed the highest sensitivity (0.78), specificity (0.94), accuracy (0.91), and AUC value of 0.83. The conclusion is that the ResNet10 CNN model shows good performance in conventional MRI scans and is highly applicable in clinical settings. These findings help improve the diagnostic accuracy of conventional MRI in differentiating glioma recurrence and radiation necrosis.

## 6 Convolutional neural networks: challenges and future prospects in the field of brain tumors

Although CNN has made remarkable progress in brain tumors, it still faces some challenges. Firstly, the data of rare brain tumor cases are scarce, which leads to the poor performance of the model in distinguishing minority tumors; Secondly, the heterogeneity of image data (differences in equipment, parameters and scanning sequence) will still affect the cross-center generalization ability of the model (37); Finally, the “black box” characteristics of the model lead to the lack of interpretability of the diagnosis results, and it is difficult to gain the wide trust of clinicians (38). Convolutional neural network (CNN) is reshaping the clinical practice and research paradigm of brain tumor science with the power of technological innovation. Its future prospect focuses on three directions: accuracy, integration and generalization, which is expected to completely change the pattern of brain tumor diagnosis and treatment. At the diagnostic level, CNN will break through the limitation of single mode and build a multi-dimensional fusion framework of “image-gene-clinic” (39). By integrating MRI multi-sequence data and genomic characteristics, the noninvasive prediction of molecular markers such as IDH mutation and 1p/19q co-deletion was realized, and the accuracy was further improved on the basis of the existing AUC value of 0.92, replacing some invasive biopsies. At the same time, it can be explained that the integration of AI (XAI) technology will solve the “black box” problem, and with the help of Grad-CAM attention heat map and other tools, the model can directly mark suspicious lesions and enhance the trust of clinicians. In the field of treatment planning, CNN will realize the leap from static evaluation to dynamic prediction. Combined with longitudinal image data and continuous learning algorithm, the model can capture the tumor evolution trajectory and false progress risk in advance, and provide real-time guidance for accurate delineation of radiotherapy target area and determination of surgical boundary (40). The landing of augmented reality surgical navigation can project the segmentation results of CNN to the surgical field of vision in real time, which can protect the functional brain area while maximizing the tumor resection rate and reduce the risk of recurrence (41). In the future, CNN will deeply integrate into the whole cycle management of brain tumors, from early screening and accurate classification to prognosis prediction and personalized treatment plan generation to form a closed loop. With the improvement of man–machine cooperation mechanism, this technology will break through the boundary of laboratory and become a core auxiliary tool for clinicians, which will inject continuous motivation into improving the survival rate and quality of life of patients with brain tumors (42).

## 7 Conclusion

Convolutional neural network (CNN) has shown remarkable value in the image analysis of brain tumors, and has become a key tool for auxiliary diagnosis and research. Its core advantage is that it can automatically extract layered features from multi-modal MRI and other images, effectively identify the heterogeneous features of tumors, such as irregular boundaries, necrotic areas and enhancement patterns, and achieve high-precision tumor segmentation, classification and grading. However, the clinical transformation of this technology still faces multiple challenges. First of all, the model is highly dependent on large-scale and high-quality labeled data, while the medical image data is difficult to obtain, the labeling cost is high and there are differences among experts, which may introduce bias. Secondly, the “black box” feature of CNN makes the decision-making process difficult to explain, which becomes an important obstacle in medical scenes that require high credibility. In addition, the differences of equipment and scanning protocols in different medical institutions affect the generalization ability of the model, and it is necessary to improve the accuracy through cross-center data training and standardization. The future development direction should focus on developing lightweight real-time diagnosis model to meet the needs of clinical work; and promote multi-center and prospective clinical verification to ensure the safety and reliability of the algorithm. In addition, integrating artificial intelligence into neurosurgery is the next step in the new era of medicine, which allows more personalized patient treatment and management, thus bringing better patient results (43, 44). Medical professionals agree that artificial intelligence is a clear and valuable tool for nerve intervention and surgery. Generally speaking, CNN provides a powerful tool for accurate diagnosis and treatment of brain tumors, but its comprehensive integration into clinical practice still depends on the coordinated progress of technology, data and supervision system.
